# A Novel Variant of X-Linked Moesin Gene in a Boy With Inflammatory Bowel Disease Like Disease-A Case Report

**DOI:** 10.3389/fgene.2022.873635

**Published:** 2022-06-09

**Authors:** Youhong Fang, Youyou Luo, Yang Liu, Jie Chen

**Affiliations:** ^1^ Department of Gastroenterology, The Children’s Hospital, Zhejiang University School of Medicine, National Clinical Research Center for Child Health, Hangzhou, China; ^2^ Department of Pathology, The Children’s Hospital, Zhejiang University School of Medicine, National Clinical Research Center for Child Health, Hangzhou, China

**Keywords:** moesin gene, intestinal ulcer, colitis, very early-onset inflammatory bowel disease, primary immunodeficiency disease

## Abstract

Variants in the *MSN* gene were recently reported as the cause of a primary immunodeficiency disease called X-linked moesin-associated immunodeficiency (X-MAID). Hitherto, only 10 patients were reported worldwide. Here, we report a boy who presented with recurrent high fever, oral ulcers, abdominal pain, and hematochezia for over 2 weeks. His serum inflammatory markers were elevated, and colonoscopy showed multiple colon ulcers and terminal ileum ulcers which resemble colitis caused by inflammatory bowel disease. A novel heterozygous variant c.934G>T(p.Glu312Ter) in the *MSN* gene was identified using whole exome sequencing (WES) and trio analysis. Intestinal ulcers were almost healed after inducing therapy with steroids and maintenance treatment of anti-TNFα therapy. We summarized the genotype and phenotype of reported X-MAID patients and presented the patient’s unique phenotype in this study. This study also expanded the spectrum of *MSN* mutation-caused immunodeficiency.

## Introduction

Variants in the moesin (*MSN*) gene were first recognized as a cause of congenital disease in humans in 2016; the resulting disease is referred to as X-linked moesin-associated immunodeficiency (X-MAID, #300988) (Lagresle-Peyrou et al., 2016). Patients with hemizygous mutations in the *MSN* gene present with lymphopenia, hypogammaglobulinemia, and susceptibility to bacterial and viral infections (Lagresle-Peyrou et al., 2016). X-MAID patients also exhibit impaired T-cell proliferation and altered migration and adhesion capacities (Lagresle-Peyrou et al., 2016). MSN, ezrin and radixin are members of the ezrin-radixin-moesin (ERM) family. ERM is a cell-structure-related protein that regulates the actin cytoskeleton and plasma membranes (Burns et al., 2017). Although MSN, ezrin, and radixin are structurally and functionally similar, they demonstrate different physical functions and expression patterns. Ezrin protein is mostly expressed in epithelial cells, whereas MSN predominates in endothelial cells (Berryman et al., 1993). MSN and ezrin play important roles in lymphoid cell proliferation, migration and adhesion (Lagresle-Peyrou et al., 2016). *MSN* knockout mice show impaired chemotaxis of T and B lymphocytes (Hirata et al., 2012).

X-MAID is classified as a primary immunodeficiency (PID), and most PIDs are monogenic diseases. In recent years, PIDs have been reported to have a close relationship with very early-onset inflammatory bowel disease (VEOIBD), which is defined as inflammatory bowel disease (IBD) that occurs before 6 years of age. PIDs that mainly present with IBD-like features belong to monogenic IBD as well. To date, more than 70 genes have been discovered that are associated with monogenic IBD. Some genes reported as associated with immune deficiency or inborn errors of immunity. The majority (>70–80%) of VEOBD patients not have a specific identified causal genetic etiology (Charbit-Henrion et al., 2018). However, the rate of genetic discoveries among VEOIBD patients is increasing, especially in the group of infantile onset IBD patients. The well-known genes related to monogenic IBD are *IL10*/*IL10R*, *XIAP* and *CGD*. Among these genes, the most common variants that lead to VEOIBD are *IL10R* mutations. The main mechanisms related to monogenic IBD include intestinal epithelial barrier function, phagocytic bactericidal activity, hyper- or autoimmune inflammatory pathways, and the development and function of the adaptive immune system (Kelsen et al., 2020). The relationship between PIDs and IBD-like disease is not completely known. Variants of the *MSN* gene have not been reported to be associated with intestinal colitis. Here, we report a VEOIBD patient with a novel variant of the X-linked *MSN* gene.

## Case Description

**FIGURE 1:**
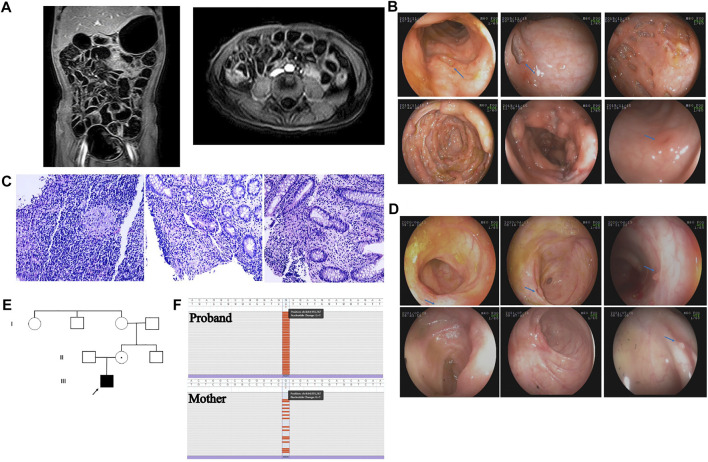
**(A)** Abdominal CT showed irregular wall thickness and enhancement of ascending colon and terminal ileum wall; enlargement of abdominal lymph nodes **(B)** Colonoscopy detected multiple ulcers in the terminal ileum, colon; and scatter ulcers in the rectum. The ulcers had a sharp margin, and most had no exudate or edema, mainly located at ascending colon and cecum. **(C)** Histological study of the mucosal biopsy indicated segmental chronic active inflammation of the colon and terminal ileum. Cryptitis, crypt abscess and noncaseating granulomas was observed in the ileum and colon **(D)** Repeated colonoscopy showed remarkably improvement of intestinal inflammation. **(E)** A novel nonsense heterozygous mutation of c.934G>T (p.Glu312Ter) was identified in the *MSN* gene. The mutation of the proband was inherited from his mother. **(F)**.

A boy aged 5 years and 2 months presented with recurrent high fever for 18 days, accompanied by a mild cough, abdominal pain, diarrhea and hematochezia. Although he was actively treated with intravenous antibiotics and immunoglobin, his fever was persistent. He was the first child in his family. A single episode of mild pneumonia had occurred when he was 4 months old and he received intravenous antibiotics for several days. There was no history of recurrent respiratory infection or eczema. No history of prolonged umbilical stump as well. He did not have chickenpox and was not vaccinated against this. On physical examination, he had moderate malnutrition, multiple ulcers in the oral cavity and on the surface of the tongue, and no lymphadenopathy or hepatosplenomegaly. Laboratory examinations revealed obvious elevation of inflammatory markers; the white blood cell count was 19.44 × 10^9^/L, of which neutrophils accounted for 66.4%; the C-reactive protein (CRP) level was 17.34 mg/L; the erythrocyte sedimentation rate (ESR) was 73 mm/h; and the serum procalcitonin level was 0.412 ng/ml. In addition, the patient had moderate anemia, with a hemoglobin level of 84 g/L, and his serum albumin level was decreased, at 27.4 g/L. Serum antibodies against cytomegalovirus (CMV) and Epstein–Barr virus (EBV) were negative, and a T-SPOT TB test was negative. Number of CD19^+^ B lymphocytes was reduced, accounting for 16.45% (reference range (r.r): 18.5–28.0%) of lymphocytes; CD3^+^ T lymphocytes accounted for 54.1% (r.r: 56.0–68.0%) of lymphocytes, CD4^+^ T lymphocytes accounted for 31.45% (r.r: 29.0–40.0%), and CD8^+^ T lymphocytes accounted for 18.35% (r.r: 19.0–25.0%). The ratio of CD4^+^ to CD8^+^ T cells was normal, 1.71 (r.r: 1.1–2.0/1). Abdominal computed tomography scan showed irregular wall thickness, enhancement of the ascending colon and terminal ileum wall, and enlargement of the abdominal lymph nodes (Figure 1A). Intestinal ultrasound showed that the walls of the terminal ileum and colon were thickened. The inflammation affected the vermiform appendix. Colonoscopy was performed, and multiple ulcers in the terminal ileum and colon were detected. The area of the largest ulcer was approximately 3 cm × 4 cm. The ulcers had sharp margins, and most had no exudate or edema; these lesions were mainly located in the ascending colon and cecum. There were also scattered ulcers located in the rectum **(** Figure 1B). Histological study of a mucosal biopsy indicated segmental chronic active inflammation of the colon and terminal ileum, cryptitis and crypt abscess were observed at the colon; additionally, several noncaseating granulomas was observed in the ileum and descending colon (Figure 1C). Microbial metagenomic next-generation sequencing (mNGS) of the intestinal biopsy did not reveal any opportunistic pathogens. The patient was diagnosed with VEOIBD and was treated with 2 mg/kg intravenous methylprednisolone twice a day. His fever subsided on the second day of the intravenous methylprednisolone regimen. Then, he was switched to oral prednisone, which was gradually tapered down within 3 months. Repeated colonoscopy showed that the intestinal inflammation was remarkably improved. Only small ulcers in the terminal ileum were observed, and the ulcers located in the colon had healed, leaving a scattering of small inflammatory polyps (Figure 1D). Capsule endoscopy confirmed terminal ileal ulcers. The patient took oral mercaptopurine for 4 months. Although he had a wild-type genotype for thiopurine methyltransferase (*TPMT*) and *NUDT15*, he experienced recurrent neutropenia. He was switched to methotrexate (MTX) due to neutropenia, and he had severe sepsis when he was on MTX. He presented with high fever, chill, delirium and shock, accompanied with remarkably elevated CRP and procalcitonin levels. He was treated with vasoactive drug and intravenous antibiotics in the intensive care unit for 1 week. Because of nonspecific intestinal colitis and early onset of disease, he was suspected to have monogenic IBD. The repeated immune work of the peripheral blood was showed in Supplementary Table S1. The number and percentage of memory B cells, central CD8^+^ T cells, Th1 cells and NK cells were obviously reduced.

## Genetic Analysis

The patient and his family underwent whole exome sequencing (WES) and trio analysis due to the early onset of intestinal disease. Peripheral blood samples from the patient and his parents were collected and subjected to next-generation sequencing (NGS) at Running Gene Inc. (Beijing, China). In brief, DNA was isolated from peripheral blood using a DNA Isolation Kit (Blood DNA Kit V2, CW2553). For all samples, shearing worked very consistently, and the peak of the size distribution was approximately 200 bp. DNA libraries were prepared with a KAPA Library Preparation Kit (Kapa Biosystems, KR0453) following the manufacturer’s instructions. The libraries were estimated with a Qubit dsDNA HS Assay kit (Invitrogen, Q32851). Array capture, hybridization of pooled libraries to the capture probes and removal of nonhybridized library molecules were carried out according to the SeqCap hybrid mix system. Sequencing, sample dilution, flow cell loading and sequencing were performed according to the Illumina specifications. DNA libraries were sequenced on the Illumina Nova Seq platform as paired-end 200-bp reads. Variant calling and annotation quality control were applied to raw data (stored in FASTQ format) obtained from NovaSeq to guarantee the meaningfulness of downstream analysis. High-quality paired-end reads were aligned to the human reference genome sequence from the UCSC database (build 37.1 version hg19, http://genome.ucsc.edu/) using the Burrows–Wheeler Aligner (https://github.com/lh3/bwa). We estimated quality scores and performed consensus SNP and insertion and deletion (indel) calling using the Genome Analysis Tool Kit. All the called variants were annotated using several public databases (1000 Genomes Project, ExAC, gnomAD, ESP6500, CCDS, RefSeq, Ensembl, etc.). Candidate variants were classified according to the American College of Medical Genetics and Genomics (ACMG) guidelines (Richards et al., 2015).

A novel hemizygous mutation, c.934G>T (p. Glu312Ter), was identified in the *MSN* gene. The pathogenic variant in the proband was inherited from his mother (Figures 1E,F). The variant meets PVS1 and PM2, and therefore was likely pathogenic according to ACMG guideline. This pathogenic variant was located in the coiled coil domain of the *MSN*. It was a truncation mutation that resulted in the pre-termination of MSN protein. We specially screened variants that associated with IBD as well as PID, and we did not discover any suspected variant. We hypothesize that this pathogenic variant affects the anchor of the MSN protein and prevents it from playing its role in regulating the actin cytoskeleton in the relevant cells.

## Discussion

Variants in the *MSN* gene were recently reported as the cause of a primary immunodeficiency disease called X-MAID by Chantal Lagresle-Peyrou *et al.* in 2016 (Lagresle-Peyrou et al., 2016). They investigated 7 male patients from 5 different families who had recurrent bacterial and viral infections, especially varicella-zoster virus (VZV) infections (Lagresle-Peyrou et al., 2016). Subsequently, Ottavia M. Delmonte reported a severe combined immunodeficiency patient who was identified by newborn screening and whose *MSN* mutation was sequenced by WES at 25 months of age (Delmonte et al., 2017). In a 24-year-old man with confirmed immunodeficiency of unknown cause, Gabrielle Bradshaw identified a variant of *MSN* by WES and whole-genome microarray copy number variant (CNV) analysis (Bradshaw et al., 2018). HUI Xiao-ying reported a similar case in a Chinese boy in 2017 (Xiao-chuan et al., 2017). Sarah E. Henrickson et al. (2019) reported three cases of X-MAID who were presented with severe combined immunodeficiency (SCID) and treatment with HSCT. The laboratory features of X-MAID are reduced lymphocytes, low immunoglobin and fluctuating neutropenia (Lagresle-Peyrou et al., 2016). There have been only five articles on this disease worldwide, describing a total of 12 patients from nine families; furthermore, there is a ESID registry study calling for an international, multicenter, retrospective study of patients with X-MAID, and they have collected about a total of 16 patients. Here, we summarized the clinical and genetic mutation features of reported X-MAID patients along with those of our case in Table 1. All the patients were male, and the age of genetic diagnosis ranged from newborn to 69 years, with median age of 5 years. The clinical manifestations were recurrent bacterial and viral infections of the respiratory and gastrointestinal systems. Eight patients had skin manifestations, mainly including eczema and molluscum contagiosum. Common immune-associated laboratory features of reported X-MAID are summarized in Table 2, including low neutrocyte levels (11/13), low levels of lymphocytes (12/13), reduced CD19^+^ B lymphocytes (13/13), reduced CD4^+^ (13/13) and CD8^+^ T cell counts (12/13), and low levels of IgG (8/10). While our patient presented a different phenotype. He did not have obvious recurrent infections till now, and the predominant clinical feature was IBD-like intestinal inflammatory.

**TABLE 1:** The clinical and genetic mutation features of those reported in literatures along with our case.

	Gene mutation	Mutation derived	Age of VZV infection, years	Age of evaluation, years	Other infections	Skin manifestations and others	Treatment
P1	c.511C>T p.(Arg171Trp)	Mother	3.5	18	Lung infections	Contagiosum molluscum, Eczema	IVIG, SMZ
P2	c.511C>T p.(Arg171Trp)	Mother	1.2	9	Lung infections, bronchitis, gastroenteritis, recurrent severe acute otitis	Contagiosum molluscum, Eczema, alopecia, seborrheic dermatitis	IVIG, SMZ, G-CSF
P3	c.511C>T p.(Arg171Trp)	Mother	1.2	4	Gastroenteritis, *Chryseobacterium* species infection	TTP, cerebrovascular stroke	IVIG, SMZ
P4	c.511C>T p.(Arg171Trp)	NA	1.2	5	NE	Eczema, ecthyma	IVIG, G-CSF, HSCT
P5	c.511C>T p.(Arg171Trp)	Mother	-	3.5	Recurrent gastroenteritis, recurrent upper respiratory tract infections	Eczema, alopecia, Gibert Disease	IVIG, SMZ
P6	c.511C>T p.(Arg171Trp)	NA	5	39	Severe pneumonia with septic shock, recurrent respiratory and urinary tract infections	Contagiosum molluscum, eczema	-
P7	c.1657C>T p.(Arg553Ter))	NA	<10	69	Lung infections bronchitis, recurrent sinopulmonary infections	-	Azithromycin
P8	c.511C>T p.(Arg171Trp)	De novo	-	2.25	Parainfluenzae virus type 2 infection, Oral thrush; enteroditis Rhino/Entero virus infection	Atopic dermatitis	IVIG
P9	c.511C>T p.(Arg171Trp)	Mother	-	8	Recurrent respiratory infections, diarrhea	Eczema, ecthyma	IVIG, SMZ
P10	c.511C>T p.(Arg171Trp)	Mother	1.5	24	Bronchitis, repeated upper respiratory tract infections, ear infections, periorbital cellulitis, pneumonia	Skin infections, paronychiae, thrombocytopenia	IVIG, G-CSF
P11	c.511 C>T p.(Arg171Trp)	-	-	0.17	Diarrhea, CMV infection	-	SMZ/trimethoprim sulfate, fluconazole, IVIG, HSCT
P12	c.511 C>T p.(Arg171Trp)	-	-	Newborn	-	-	SMZ/trimethoprim sulfate, fluconazole, IVIG, HSCT
Our case	c.934G>T p.(Glu312Ter)	Mother	-	5	Fever, pneumonia, sepsis	Enteropathy	IVIG, steroid, IFX

VZV, varicella-zoster virus; TTP, thrombotic thrombocytopenic purpura; IVIG, intravenous immunoglobin; SMZ, sulfamethoxazole; G-CSF, granulocyte-colony stimulating factor; HSCT, hematopoietic stem cell transplantation; CMV, cytomegalovirus; IFX, infliximab.

**TABLE 2:** The immune associate results of reported X-MAID patients and our case.

	P1	P2	P3	P4	P5	P6	P7	P8	P9	P10	P11	P12	This case
Neutrocytes	L	L	L	L	L	L	N	L	L	N	L	L	L
Monocytes	L	L	L	N	L	L	L	L	L	N	ND	ND	L
Lymphocytes	L	L	L	L	L	L	L	L	L	L	L	L	N
CD19^+^ cells	L	L	L	L	L	L	L	L	L	L	L	L	L
Naive B cells	L	L	L	ND	ND	ND	ND	ND	N	ND	ND	ND	L
Natural killer cells	L	L	L	L	L	L	L	ND	ND	L	N	L	L
CD4^+^ cells	L	L	L	L	L	L	L	L	L	L	L	L	L
CD8^+^cells	L	L	L	L	L	N	L	L	L	L	L	L	L
Naive CD4^+^T cells	L	L	L	ND	L	L	L	N	L	ND	ND	ND	N
Naive CD8^+^T cells	L	L	L	ND	L	L	L	L	N	ND	ND	ND	N
CD57^+^CD8^+^ T cells	H	H	H	ND	H	H	H	ND	ND	ND	ND	ND	N
IgG	L	L	L	L	NE	L	L	L	L	N	ND	ND	N
IgA	L	N	L	L	L	L	N	N	L	L	ND	ND	N
IgM	L	L	L	L	L	L	L	L	L	L	ND	ND	N
IgE	NE	N	NE	N	N	NE	NE	N	N	ND	ND	ND	N

L, low; H, high; N, normal; NE, Non-evaluated; ND, not determined; Ig, immunoglobin.

Although *MSN* mutation affected both T cell and B cell function, patients with X-MAID presented with deficiencies in cellular immunity and humoral immunity. These patients had a relatively good prognosis. The oldest patient was 69 years old, and the most severe condition was severe pneumonia accompanied by shock. Other complications were common infections and were treated with immunoglobin infusion and antibiotics. Sulfamethoxazole (SMZ) tablets and recombinant human granulocyte colony-stimulating factor (G-CSF) were used to prevent opportunistic infections for recurrent neutropenia. Three patients exhibited severe combined immunodeficiency received hematopoietic stem cell transplantation (HSCT). However, mutations in the *MSN* gene that can lead to IBD-like feature have not been reported, and the optimal treatment of colitis caused by *MSN* variant is not clear. Remission of inflammatory colitis was induced with steroid and maintained with anti-TNF therapy in this case. There is no standard treatment for VEOIBD or IBD-like features caused by immunodeficiencies, the main treatment included supporting treatment, symptomatic treatment and conventional treatment for IBD, such as steroids, immunosuppressants, biological treatment and thalidomide, et al. The patient in our case had persistent fever, no chronic history of disease, and the endoscopy and pathological finding of biopsy indicated the feature of IBD, so we used steroid to induce remission. The patient did not have allergy history, and his nutrition status was normal, so he did not have any special diet.

The human moesin gene, *MSN,* is located on chromosome Xq12 and has 13 exons distributed over more than 30 kb. Only two different variants of the *MSN* gene have been reported. Eleven patients were identified to harbor the same variant of c.511C>T (p. Arg171Trp), which is located in the FERM domain. Another reported variant is c.1657C>T (p.Arg553Ter), which is located in the F-actin binding domain. Our case has a nonsense variant of c.934G>T (p.Glu312Ter) located in the coiled coil domain. This variant alters the codon for Glu312 to a termination codon, which causes premature termination of MSN protein synthesis. Our case is the second reported nonsense variant in *MSN* gene, and the phenotype is differed from other pathogenic variants, which may suggest that nonsense variants in *MSN* may cause IBD-like features. The variant of c.934G>T (p.Glu312Ter) is classified as a variant of likely pathogenic according to the ACMG guidelines (Richards et al., 2015). The pLi score for *MSN* in gnomAD is 1, which means the gene is highly intolerant of loss of function variants (Ziegler et al., 2019), this adds further evidence that *MSN* variants cause immunodeficiency and widen the reported phenotypes.

MSN protein is mainly expressed in endothelial cells, lymphocytes and platelets, and it regulates the actin cytoskeleton. *MSN* knockout mice was observed to impair the migration and adhesion of lymphocyte cells; naive CD8^+^ T cells were affected most. Recently, isolated CD8^+^ Treg cells from moesin-deficient mice exhibited impaired proliferation in response to IL-15, which shows the importance of moesin in IL-15–dependent CD8^+^ Treg cell homeostasis (Satooka et al., 2017). Additionally, moesin regulates NK cell survival through IL-15–mediated signaling *in vivo* (Satooka et al., 2021). Altogether, *MSN* mutation may affect both innate and adaptive immunity *in vivo*. However, the underlying mechanism by which variants of *MSN* causes IBD-like features needs further investigation.

PIDs have a cross-connection with VEOIBD. The actin cytoskeleton is required for many immune cell functions, including migration, adhesion, phagocytosis, assembly of complex intercellular contacts, and cell division (Burns et al., 2017). PID-associated actin regulatory defects include Wiskott-Aldrich syndrome (WAS), WASp-interacting protein (WIP) deficiency, *DOCK2* and *DOCK8* deficiencies, moesin deficiency, leukocyte adhesion defects (LAD1, 2, 3 and IV) and megakaryoblastic leukemia 1 (*MKL1*), and these defects lead to combined immunodeficiencies or phagocyte disorders (Burns et al., 2017). It has been reported that WAS can present as IBD-like intestinal inflammation (Ohya et al., 2017). We suspect that the mechanism of *MSN*-associated colitis is as follows. First, similar to WAS, MSN is a member of the cell-structure-related proteins that regulates the cell actin cytoskeleton and plasma membrane. The actin cytoskeleton is required for many immune cell functions. Dysfunction of immune cells is related to nonspecific colitis or IBD-like features. Secondly, MSN is dominantly expressed on lymphocytes, which plays an important role in the regulation of intestinal inflammation. Chronic intestinal inflammation could lead to IBD-like features. In our case, the boy exhibited severe intestinal inflammation at first, a decrease in B lymphocytes and neutrocytes, low albumin, and elevation of CRP and ESR, and he was not responsive to intravenous antibiotics treatment. The negative result of blood serologic tests, immunohistochemistry study and the mNGS test of the mucosal biopsy did not indicate any infection that caused the inflammation of the colon. The WES did not reveal any causative variant that related to PID or VEOIBD. After the administration of steroid, the inflammatory markers and intestinal inflammation were obviously improved, which indicated that an immune mechanism participated in the process of inflammation. However, other typical phenotypes of X-MAID patients, such as hypogammaglobulinemia, neutropenia, VAZ infection, recurrent respiratory tract infections, and eczema, were not obvious in this patient. The mechanism of intestinal inflammation in this patient is not clear, but we presume that it may be associated with dysregulation of immune cells and altered functioning of lymphocytes. Intriguingly, some immunocompromising diseases, such as common variable immunodeficiency, hyper-IgM syndrome, and selective IgA deficiency, present with IBD-like intestinal inflammation (Albshesh et al., 2021). In an immunocompromised patient, opportunistic infections, such as cytomegalovirus or Epstein-Barr virus infection, may contribute to intestinal inflammation as well. However, this patient did not have recurrent infections, and his pathogen tests were negative. The mNGS of an intestinal biopsy was negative as well. The patient’s colon ulcers were almost completely healed after steroid treatment, which may indicate that the ulcers were induced by immune dysregulation. He could not tolerate mercaptopurine or MTX, so he was ultimately switched to IFX to induce and maintain mucosal healing. The intestinal ulcers were healed after three doses of infused IFX.

In conclusion, we reported a patient with nonsense variant of *MSN* who presented with IBD-like features. This is the first reported case of an X-MAID patient maily presented with enteropathy. The pathogenesis of intestinal colitis is not clear and requires further basic medical research.

## Data Availability

The datasets for this article are not publicly available due to concerns regarding participant/patient anonymity. Requests to access the datasets should be directed to the corresponding author.

## Supplementary Material

Supplementary Table S1 The number and percentage of peripheral blood immune cells of the patient.
