# Supplementary material for: A Novel Variant of X-Linked Moesin Gene in a Boy With Inflammatory Bowel Disease Like Disease-A Case Report
Source: Front Genet. 2022 Jun 9;13:873635. doi: 10.3389/fgene.2022.873635 (PMC9224403; doi:10.3389/fgene.2022.873635)
Supplement: Supplementary file 1 [file Table1.docx]

Supplement table 1. The number and percentage of peripheral blood immune cells of the patient

| Peripheral blood immune cells | Number (/μl) | Percentage (%) |
| --- | --- | --- |
| Naive B cells | 61.05(r.r: 79.27-175.1) | 76.57 (r.r 64.21-87.32) of total B lymphocyte cells |
| Memory B cells | 2.16 (r.r: 8.34-34.76) | 1.72 (r.r:15.94-55.64) of total B cells |
| Central CD4^+^ | 82.79 (r.r:106.2-196.9) | 8.46 (r.r:7.19-26.33) of total T lymphocyte cells |
| Central CD8^+^ T cells | 1.53 (r.r: 4.909-12.64) | 0.16 (r.r: 0.37-1.63) of total T lymphocyte cells |
| Th1 cells | 56.58(r.r: 89.88-140.7) | 9.83 (r.r:12.65-36.24) of CD4^+^ T cells |
| NK cells | 21.03 (r.r:150-1100) | 3.88 (r.r:7.00-40.00) of lymphocyte cells |
